# Calcium-binding proteins are altered in the cerebellum in schizophrenia

**DOI:** 10.1371/journal.pone.0230400

**Published:** 2020-07-08

**Authors:** Francisco Vidal-Domènech, Gemma Riquelme, Raquel Pinacho, Ricard Rodriguez-Mias, América Vera, Alfonso Monje, Isidre Ferrer, Luis F. Callado, J. Javier Meana, Judit Villén, Belén Ramos

**Affiliations:** 1 Psiquiatria Molecular, Institut de Recerca Sant Joan de Déu, Esplugues de Llobregat, Spain; 2 Dept. de Bioquímica i Biologia Molecular, Facultat de Medicina, Universitat Autònoma de Barcelona, Bellaterra, Spain; 3 Department of Genome Sciences, School of Medicine, University of Washington, Seattle, Washington, United States of America; 4 Parc Sanitari Sant Joan de Déu, Sant Boi de Llobregat, Spain; 5 Departamento de Patologia y Terapeutica Experimental, Universidad de Barcelona, Senior consultant Servicio Anatomia Patológica, Hospital Universitario de Bellvitge-IDIBELL, CIBERNED, Hospital de Llobregat, Barcelona, Spain; 6 Department of Pharmacology, University of the Basque Country UPV/EHU, Leioa, Bizkaia, Spain; 7 Centro de Investigación Biomédica en Red de Salud Mental, Madrid, CIBERSAM, Spain; 8 Biocruces Bizkaia Health Research Institute, Barakaldo, Spain; Medical University of Lodz, POLAND

## Abstract

Alterations in the cortico-cerebellar-thalamic-cortical circuit might underlie the diversity of symptoms in schizophrenia. However, molecular changes in cerebellar neuronal circuits, part of this network, have not yet been fully determined. Using LC-MS/MS, we screened altered candidates in pooled grey matter of cerebellum from schizophrenia subjects who committed suicide (n = 4) and healthy individuals (n = 4). Further validation by immunoblotting of three selected candidates was performed in two cohorts comprising schizophrenia (n = 20), non-schizophrenia suicide (n = 6) and healthy controls (n = 21). We found 99 significantly altered proteins, 31 of them previously reported in other brain areas by proteomic studies. Transport function was the most enriched category, while cell communication was the most prevalent function. For validation, we selected the vacuolar proton pump subunit 1 (VPP1), from transport, and two EF-hand calcium-binding proteins, calmodulin and parvalbumin, from cell communication. All candidates showed significant changes in schizophrenia (n = 7) compared to controls (n = 7). VPP1 was altered in the non-schizophrenia suicide group and increased levels of parvalbumin were linked to antipsychotics. Further validation in an independent cohort of non-suicidal chronic schizophrenia subjects (n = 13) and non-psychiatric controls (n = 14) showed that parvalbumin was increased, while calmodulin was decreased in schizophrenia. Our findings provide evidence of calcium-binding protein dysregulation in the cerebellum in schizophrenia, suggesting an impact on normal calcium-dependent synaptic functioning of cerebellar circuits. Our study also links VPP1 to suicide behaviours, suggesting a possible impairment in vesicle neurotransmitter refilling and release in these phenotypes.

## Introduction

Schizophrenia constitutes a complex disorder with a mixture of symptoms and cognitive deficiencies. It is considered a brain disorder in which a malfunction of multiple regions in distributed brain macro- and microcircuits could lead to the different symptoms. According to the hypothesis of cognitive dysmetria, which is based on neuroimaging findings, the alteration of specific components of the cortico-cerebellar-thalamic-cortical circuit could contribute to the impairment of mental coordination processes, leading to the emergence of symptoms in schizophrenia [1–3]. The cerebellum, as part of this circuit, has been suggested to play a role in the production of the range of symptoms and cognitive impairments in schizophrenia. Much evidence has been collected over the last few decades supporting the involvement of the cerebellum in higher cognitive functions and in the pathophysiology of this neurodevelopmental disorder [4–6]. Based on the clinical signs of individuals with cerebellar lesions and functional neuroimaging studies in patients with schizophrenia, it has been suggested that the impairment of the cerebellar function could affect many altered domains in schizophrenia, including executive functions, working memory, language, attention, social cognition and emotion (reviewed in [4,6,7]). The lateral cerebellar cortex is involved in some of these cognitive abilities, such as working memory, executive functions and speech [5,8–10]. The complex cytoarchitecture of the cerebellar cortex comprises a variety of neurons including GABAergic neurons, such as Purkinje cells, basket cells, stellate cells, and Golgi cells, and glutamatergic cells, such as granule neurons and unipolar brush cells. Both Purkinje cells and granule cells are key in orchestrating the organisation of this circuitry during development, and thus defective regulation of the intracellular mechanisms maintaining the normal synaptic functioning of these cells could impact on internal cerebellar circuit activities and the cerebellum output signal to the cortex. These altered cerebellar circuits have been linked to a number of neurological and psychiatric conditions such as schizophrenia (reviewed in [11,12]). Moreover, molecular hypothesis-driven, studies have found genes from the GABAergic and glutamatergic neurotransmission system with altered expression in the cerebellar cortex, supporting the idea that the regulation of these systems is also disrupted in the cerebellum [13–18]. Gene expression changes have been detected in the cerebellum using transcriptomic screenings in a few studies in schizophrenia [19–21]. However, to the best of our knowledge, proteomic studies to identify altered proteins in cerebellar neuronal circuits have not yet been performed. The cerebellar cortex is a highly homogeneous brain area composed mainly of cerebellar granule neurons. This characteristic makes the cerebellar cortex an attractive area for investigating proteomic changes in a relatively small sample.

Here, we conducted an exploratory quantitative proteomic screen using pooled grey matter of the cerebellar cortex from schizophrenia (1 pool of 4 samples) and control healthy individuals (1 pool of 4 samples) with the aim of identifying consistently altered proteins in schizophrenia (SZ) (S1A Fig). We then validated three selected candidates by immunoblot in a total of 47 subjects, which included the same samples from the proteomic analysis, an extended cohort (control, n = 7; SZ, n = 7) with a non-schizophrenia suicide group (n = 6), and a larger independent cohort with non-suicidal chronic schizophrenia subjects (control, n = 14; SZ, n = 13) (S1A Fig). Our validation focused on three selected candidates with a possible relevant role in the disorder and detected with robust changes in the screening.

## Materials and methods

### Brain tissue samples

For the proteomic analysis, we used post-mortem human brain tissue obtained from the *UPV/EHU brain collection*, from the cerebellum of subjects with paranoid schizophrenia who had committed suicide (n = 4) and control subjects who had died in a traffic accident and who had had no history of psychiatric episodes (n = 4) (Table 1 and S1 Table). Samples were obtained at autopsies in the Basque Institute of Legal Medicine, Bilbao, Spain, in compliance with the policies of the research and ethical boards for post-mortem studies. Toxicological screening for antipsychotics, antidepressants, and other drugs was performed at the National Institute of Toxicology, Madrid, Spain. All deaths were subjected to retrospective analysis for previous medical diagnosis. Subjects with ante-mortem criteria for paranoid schizophrenia according to the Diagnostic and Statistical Manual of Mental Disorders (DSM-IIIR and DSM-IV) were matched to control subjects who had died from accidental causes in a paired design, based on gender, age, and post-mortem delay (PMD).

**Table 1:** Demographic, clinical and tissue-related features of cases of Cohorts I and II.

**Cohort I: Pilot Validation analysis (n = 20)**							
	Schizophrenia^a^ (n = 7)	Non-schizophrenia suicide (n = 6)	Healthy Control (n = 7)	SZ-C		SZ-Suicide	
				Statistic	p-value	Statistic	p-value
Gender				N/A	^b^1.000	N/A	^b^1.000
Female	14% (n = 1)	17% (n = 1)	14% (n = 1)				
Male	86% (n = 6)	83% (n = 5)	86% (n = 6)				
Age (years)	44 ± 11	46 ± 11	45 ± 11	^c^22.00	0.782	^c^21.00	1.000
PMD (hours)	9.00 ± 3.37	9.00 ± 4.36	11.33 ± 8.36	^c^21.00	0.683	^c^19.50	0.858
pH	6.66 ± 0.60	6.90 ± 0.59	6.53 ± 0.42	^c^19.50	0.556	^c^19.50	0.882
Toxicology				N/A	N/A	N/A	N/A
Atypical AP	42.8% (n = 3)	16.7% (n = 1)	N/A				
Other	28.6% (n = 2)	50.0% (n = 3)	57.1% (n = 4)				
Drug-free	28.6% (n = 2)	33.3% (n = 2)	42.8% (n = 3)				
Somatic Disorders				N/A	N/A	N/A	N/A
Diabetes	14.3% (n = 1)		14.3% (n = 1)				
Arterial hypertension	14.3% (n = 1)		14.3% (n = 1)				
Other		16.7% (n = 1)	14.3% (n = 1)				
**Cohort II: Extended Validation cohort (n = 27)**							
	Non-suicide Schizophrenia (n = 13)		Healthy Control (n = 14)	Statistic		p-value	
Gender							
Male	100% (n = 13)		100% (n = 14)	N/A		N/A	
Age (years)	72 ± 9		70 ± 11	0.72; 25^d^		0.478	
PMD (hours)	5.62 ± 2.34		5.46 ± 1.82	0.02; 25^d^		0.984	
pH	6.88 ± 0.49		6.61 ± 0.63	1.25; 25^d^		0.223	
SZ diagnosis			N/A	N/A		N/A	
Chronic residual	69.2% (n = 9)						
Chronic paranoid	15.4% (n = 2)						
Chronic disorganized	7.7% (n = 1)						
Chronic catatonic	7.7% (n = 1)						
Age of onset of SZ (years)	22 ± 8		N/A	N/A		N/A	
Duration of illness (years)	51 ± 9		N/A	N/A		N/A	
Daily *AP* dose (mg/day)^e^	562.92 ± 514.13		N/A	N/A		N/A	
Atypical AP	7.7% (n = 1)		N/A	N/A		N/A	
Typical AP	92.3% (n = 12)		N/A	N/A		N/A	
Somatic Disorders							
Diabetes	7.7% (n = 1)		28.6% (n = 4)	N/A		0.326^b^	
Arterial hypertension	15.4% (n = 2)		57.1% (n = 8)	N/A		0.046^b^	
Dyslipidemia	7.7% (n = 1)		35.7% (n = 5)	N/A		0.165^b^	
Others	15.4% (n = 2)		7.1% (n = 1)	N/A		N/A	

Mean ± standard deviation or relative frequency are shown for each variable; PMD, post-mortem delay between death and brain sample collection; SZ, schizophrenia; C, Healthy control group; AP, antipsychotics; N/A, not applicable.

^a^Paranoid schizophrenia (n = 7).

^b^Frequencies were analysed using Fisher’s exact test.

^c^Mann-Whitney U is shown for non-parametric variables.

^d^T-statistic and degrees of freedom are shown for parametric variables.

^e^Last chlorpromazine equivalent dose was calculated based on the electronic records of drug prescription of the patients.

To validate the candidates identified in the quantitative proteomic assay, we also used an extended cohort from the *UPV/EHU brain collection* for individual sample analysis of human post-mortem cerebellum (Table 1, Cohort I). A total of 20 brains of subjects who had committed suicide (n = 12), died in an accident (n = 5) or from natural causes (n = 3) were selected. Subjects with paranoid schizophrenia (n = 7; 6 suicide victims (falling from a height (n = 4) and hanging (n = 2)) and 1 non-suicide subject that died from natural causes), control subjects (n = 7) that had died in a traffic accident (n = 5) or from natural causes (n = 2), and a non-schizophrenia suicide victim group (n = 6; falling from a height (n = 5) and hanging (n = 1)) were matched by gender, age, post-mortem delay and pH (Table 1, Cohort I). Control subjects were chosen among the collected brains on the basis, whenever possible, of the following criteria: (a) negative medical information on the presence of neuropsychiatric disorders or drug abuse, (b) accidental or natural cause of death, (c) negative results in toxicological screening for psychotropic drugs except for ethanol, and (d) a post-mortem delay not longer than 48 hours. Diagnoses were established according to the DSM-IIIR or DSM-IV. Diagnoses in the non-schizophrenia suicide victim group included obsessive compulsive disorder (n = 1), depression (n = 2), anxiety disorder (n = 1), depression and personality disorder (n = 1), and alcohol dependence (n = 1). Samples were coded by the brain collection staff to protect human subject confidentiality. The study was approved by the Institutional Ethics Committee of the Fundació Sant Joan de Déu.

For further validation analysis of protein candidates, we used a larger independent cohort of post-mortem human cerebellum of subjects with chronic schizophrenia (n = 13) who had died from natural causes and control subjects with no history of psychiatric episodes (n = 14) from the collection of neurologic tissues of Parc Sanitari Sant Joan de Déu [22] and the Institute of Neuropathology Brain Bank (HUB-ICO-IDIBELL Biobank) (Table 1, Cohort II). These collections follow the guidelines of Spanish legislation and the approval of the local ethics committees. Written informed consent was obtained from each subject. The study was approved by the Institutional Ethics Committee of Parc Sanitari Sant Joan de Déu. We matched schizophrenia and control groups by gender, age, post-mortem delay and pH. Table 1 (Cohort II) shows the demographic, clinical and tissue-related characteristics of the samples. All SZ subjects were institutionalized donors with a long duration of the illness (Table 1, Cohort II) who had no history of neurological episodes. Experienced clinical examiners interviewed each donor ante-mortem to confirm schizophrenia diagnosis according to DSM-IV and International Classification of Diseases 10 (ICD-10) criteria. The last mean daily chlorpromazine equivalent dose for the antipsychotic treatment of patients was based on the electronic records of last drug prescriptions administered up to death (Table 1, Cohort II) and was calculated as previously described [23]. The mean and standard deviation of body mass index in patients was 24.22 ± 4.26. Possible tardive dyskinesia side effect of treatments was assessed in donors using the Abnormal Involuntary Movement Scale (AIMS) [24]. The total score was calculated using the sum of items 1 to 7, which assess the severity of abnormal movements in different regions of the body. Each item was classified from 0 to 4 according to the severity (0-absence, 1-minimum, 2-mild, 3-moderate, 4-severe). The mean and standard deviation of the AIMS total score was 4.17 ± 5.22 (n = 12) with a minimum value of 0 and a maximum value of 15. 38.5% (n = 5) of the subjects showed no symptoms. 23.1% (n = 3) of the subjects showed minimum severity of symptoms, 23.1% (n = 3) mild and 7.7% (n = 1) moderate. No subjects showed severe symptoms.

Samples were coded by each brain bank or collection staff to protect human subject confidentiality.

### Protein extraction

Specimens of the lateral cerebellar cortex from the posterior lobe, extending from the pial surface to white matter and only including grey matter, were dissected from coronal slabs stored at -80 °C. Protein extracts were prepared from tissue samples using NP40 lysis buffer as described previously [25]. Protein concentration was determined by Bradford assay (Biorad).

### Mass spectrometry screening and data processing

Our screening strategy combined differential isotopic labelling of peptides via reductive dimethylation with offline fractionation by SCX and liquid chromatography coupled to tandem mass spectrometry (LC-MS/MS) on a hybrid linear ion trap orbitrap mass spectrometer (LTQ-Orbitrap)) (S1A Fig). 400 μg of total protein extracts from four pooled control (100 μg/each) and four pooled schizophrenia (100 μg/each) lysates were digested and further processed as indicated in the S1 File. Briefly, the digested peptides were dimethyl-labelled with either hydrogen (light peptides, control) or deuterium (heavy peptides, schizophrenia) isotopes through a reductive dimethylation reaction as described previously [26]. Differentially labelled peptides were then mixed 1:1. Dimethylated peptide mixtures were separated by strong cation exchange (SCX) chromatography on a polysulphoethyl A column. Peptide mixtures were analysed by LC-MS/MS. Each peptide fraction was separated by reverse phase chromatography on a capillary column and analysed online on a hybrid linear ion trap orbitrap (LTQ-Orbitrap XL, Thermo Scientific) mass spectrometer for identification and relative quantification of isotopically labelled peptide pairs. MS/MS spectra were searched against a concatenated target-decoy Uniprot human protein database (UP000005640 version 05-23-2017, n = 71,567 target sequences) using the Comet search algorithm (version 2015025) and specific search parameters (see S1 File). The mass spectrometry proteomics data have been deposited in the ProteomeXchange Consortium via the PRIDE partner repository with the dataset identifier PXD008216 [27]. Peptide matches were filtered to <1% False-Discovery Rate (FDR) and protein groups were filtered at ≥90% probability score. The log_2_ heavy/light ratio for each protein was determined and transformed to a z-score [28]. A significance value (p-value) for each protein ratio was calculated from the complementary error function for the normalized distribution of the z-scores [28]. The FDR was computed for all the p-values using the Benjamini and Hochberg method [29]. The FDR threshold was set at 0.1 for selected significant proteins with consistent changes amongst peptides. Proteins were classified according to their biological function using the Human Protein Reference Database (HPRD-http://www.hprd.org).

The altered observed proteins were compared with those previously reported in proteomic studies of other brain regions of post-mortem samples in schizophrenia based on the gene symbol.

### Immunoblotting

50 μg of total protein lysate from each sample were resolved by SDS-PAGE electrophoresis and transferred to a nitrocellulose membrane. Membranes were cut at different molecular weights and immunoblotted with rabbit polyclonal antibody against VPP1 (1:500; ab103680, Abcam) and calmodulin (1:1000; 4830, Cell Signalling Technologies), and monoclonal antibodies against parvalbumin (1:1000; MAB1572, Millipore-Chemicon) and glyceraldehyde-3-phosphate dehydrogenase (GAPDH) (1:500000; MAB374, Millipore-Chemicon) to optimize the limited available brain sample. All proteins were detected by a unique band at the predicted molecular weight with the exception of VPP1 in Cohort II, where an extra lower weight molecular band was detected; however, it was not analysed. An extra re-incubation was needed for the parvalbumin cohort I immunoblot. Densitometric quantification of candidate proteins was performed using Quantity One software (BioRad). Values were normalized to GAPDH and a control reference sample. At least two independent immunoblot analyses were performed per sample.

### Statistical analysis

For validation analysis using immunoblot we used the following procedures. Normality of the variables was assessed using the Kolmogorov-Smirnov test. Demographic and tissue-related features of the samples were compared between schizophrenia and control conditions using the Fisher exact test for qualitative variables and the unpaired Student’s t-test or the Mann-Whitney U test for quantitative parametric or non-parametric variables, respectively. The differences in protein levels between pools were analysed using the one-tailed unpaired Student’s t test, since the direction of change was expected to be the same as in the proteomics assay. The differences in protein levels between groups in the individual sample analysis were evaluated using the Kruskal-Wallis one-way analysis of variance by ranks, and the Mann-Whitney U test was used to compare differences between two groups. The Grubbs test and Pierce test were used to detect outliers for parametric or non-parametric variables, respectively; the number of outliers detected for each analysis is indicated in the figure legend. Spearman or Pearson correlation analyses were carried out to detect association of our molecular measures with other clinical, demographic and tissue-related variables (age, post-mortem delay, pH and toxicology, and daily antipsychotic dose, age of onset and duration of the illness in the SZ group of Cohort II). Statistical analysis was performed with GraphPad Prism version 5.00, with significance level set to 0.05.

## Results

### Proteomic analysis of post-mortem cerebellum from schizophrenia and control subjects

To identify proteins significantly altered in schizophrenia in the cerebellum, we analysed the proteomes of pooled protein lysates from four subjects with schizophrenia and four control subjects matched for gender, age and post-mortem delay (S1A Fig and S1 Table). We quantified 1412 proteins with a Protein Prophet probability score of more than 90% (S1 Dataset). The distribution of protein heavy-to-light (H/L) ratios revealed that some proteins of the cerebellar proteome were altered in schizophrenia (S1B and S1C Fig). We identified 99 (7%) significantly altered proteins at a false discovery rate of 10% and a protein sequence coverage greater than 5% (S2 Dataset). Moreover, 31 of the 99 significantly regulated proteins had been previously reported to be altered in proteomic analyses of other brain areas in schizophrenia (Table 2).

**Table 2:** List of altered proteins previously reported in other brain regions in proteomic studies in schizophrenia.

Brain region	Gene Symbol	Reference^a^
**DLPFC**	*GNB1*, *NDUFA12*	(Behan et al., 2009)
	*GNB1*, *MAG*	(Chan et al., 2011)
	*VIM*	(English et al., 2009)
	*GNB1*, *TF*	(Martins-de-Souza et al., 2009)
	*ATP6V0D1*, *SORBS1*, *VIM*	(Martins-De-Souza et al., 2009)
	*PNP*	(Novikova et al., 2006)
	*TF*	(Pennington et al., 2008)
	*BCL2L13*, *PHB2*, *SORBS1*, *SV2B*, *TMEM30A*	(Pinacho et al., 2016)
	*TF*	(Prabakaran et al., 2004)
	*PVALB*	(Smalla et al., 2008)
**OFC**	*DMTN*, *PGAM5*, *VDAC2*	(Velásquez et al., 2017)
**ACC**	*GNB1*, *TF*	(Clark et al., 2006)
	*AP2B1*, *ATP6V0A1*, *BAIAP2*, *CALM2*, *CCT6A*, *HSD17B4*, *NAPA*, *SRPRB*, *VDAC2*	(Föcking et al., 2015)
	*GNB1*	(Martins-de-Souza et al., 2010)
**CC**	*GNB1*, *HAPLN2*, *VIM*	(Saia-Cereda et al., 2015)
	*BAIAP2*	(Saia-Cereda et al., 2016)
	*CALM1*, *CDC42*	(Saia-Cereda et al., 2017)
**Thalamus**	*VIM*	(Martins-de-Souza et al., 2010)
**Hippocampus**	*PVALB*	(Föcking et al., 2011)
	*ACOT7*, *CCT6A*	(Schubert et al., 2015)
	*VIM*	(Nesvaderani et al., 2009)
**Temporal lobe**	*HAPLN2*	(Martins-de-Souza et al., 2009)
	*CADM1*, *CALM1*, *CDC42*, *H3F3A*, *PPP3R1*, *VIM*	(Saia-Cereda et al., 2017)
	*PHB2*, *VIM*	(MacDonald et al., 2015)

DLPFC, dorsolateral prefrontal cortex; OFC, orbitofrontal cortex; ACC, anterior cingulate cortex; CC, corpus callosum.

^a^The complete information of the references is detailed in the S1 File.

We classified the altered proteins according to their biological function and compared them to the proteins that showed no change between the two groups. Similar biological functions were found in both sets. The most prevalent function was cell communication and signalling pathways, and the transport function was enriched 10% in the non-regulated proteome and 13% in the regulated proteome (S1D Fig). We generated a list of 11 protein candidates from these functions according to two criteria: (i) the protein had been quantified with more than 4 peptides; and (ii) the protein showed a greater than 2-fold increase or decrease (Table 3).

**Table 3:** List of proteins filtered from enriched and most representative functions in the post-mortem cerebellum in schizophrenia.

Function	Acc. Number	Gene Symbol	Protein Description (Protein Symbol)	Quantified Peptides	Ratio H/L	log_2_Ratio H/L
**Transport**	**Q93050**	***ATP6V0A1***	**V-type proton ATPase 116 kDa subunit a isoform 1 (VPP1)**	5	0.09	-3.46
	A0A0A0MR02	*VDAC2*	Voltage-dependent anion-selective channel protein 2 (VDAC2)	16	0.13	-2.97
	Q86UR5	*RIMS1*	Regulating synaptic membrane exocytosis protein 1 (RIMS1)	11	0.46	-1.12
	M0R0Y2	*NAPA*	Alpha-soluble NSF attachment protein (SNAA)	6	3.90	1.97
	Q01469	*FABP5*	Fatty acid-binding protein, epidermal (FABP5)	6	5.20	2.38
**Cell Communication / Signal transduction**	**E7EMB3**	***CALM2***	**Calmodulin-2 (CALM2)**	18	2.56	1.36
	P37235	*HPCAL1*	Hippocalcin-like protein 1 (HPCL1)	9	2.64	1.40
	O00533	*CHL1*	Neural cell adhesion molecule L1-like protein (NCHL1)	17	3.01	1.59
	Q8WUD1	*RAB2B*	Ras-related protein Rab-2B (RAB2B)	6	3.17	1.66
	Q96FQ6	*S100A16*	Protein S100-A16 (S10AG)	7	3.53	1.82
	**P20472**	***PVALB***	**Parvalbumin alpha (PRVA)**	8	3.65	1.87

Access numbers from Uniprot database Uniprot; H/L ratio between heavy (schizophrenia) and light (control) peptide areas; candidates selected for validation are shown in bold.

#### Validation of protein changes in cerebellum schizophrenia samples

From this list of 11 candidates, we selected three proteins for further validation by immunoblot. We selected calmodulin 2 (CALM2), which was identified with the highest number of peptides, and two proteins with the most prominent changes in each Gene Ontology function: vacuolar proton translocating ATPase 116 kDa subunit a (VPP1) from transport, and parvalbumin alpha (PRVA) from cell communication and signal transduction (Table 3). The calmodulin antibody targets the calmodulin 1, calmodulin 2 and calmodulin 3 protein isoforms, all of which have similar molecular weight and protein sequence. We confirmed by immunoblot the changes detected by proteomics for the three candidates (suicide schizophrenia, n = 4; control, n = 4; S1 Table and S2 Fig). The fold changes of the three candidates were similar to the fold changes detected in the pooled analysis, being decreased for VPP1 and increased for calmodulin and parvalbumin (S2 Fig and Table 3).

**Fig 1:**
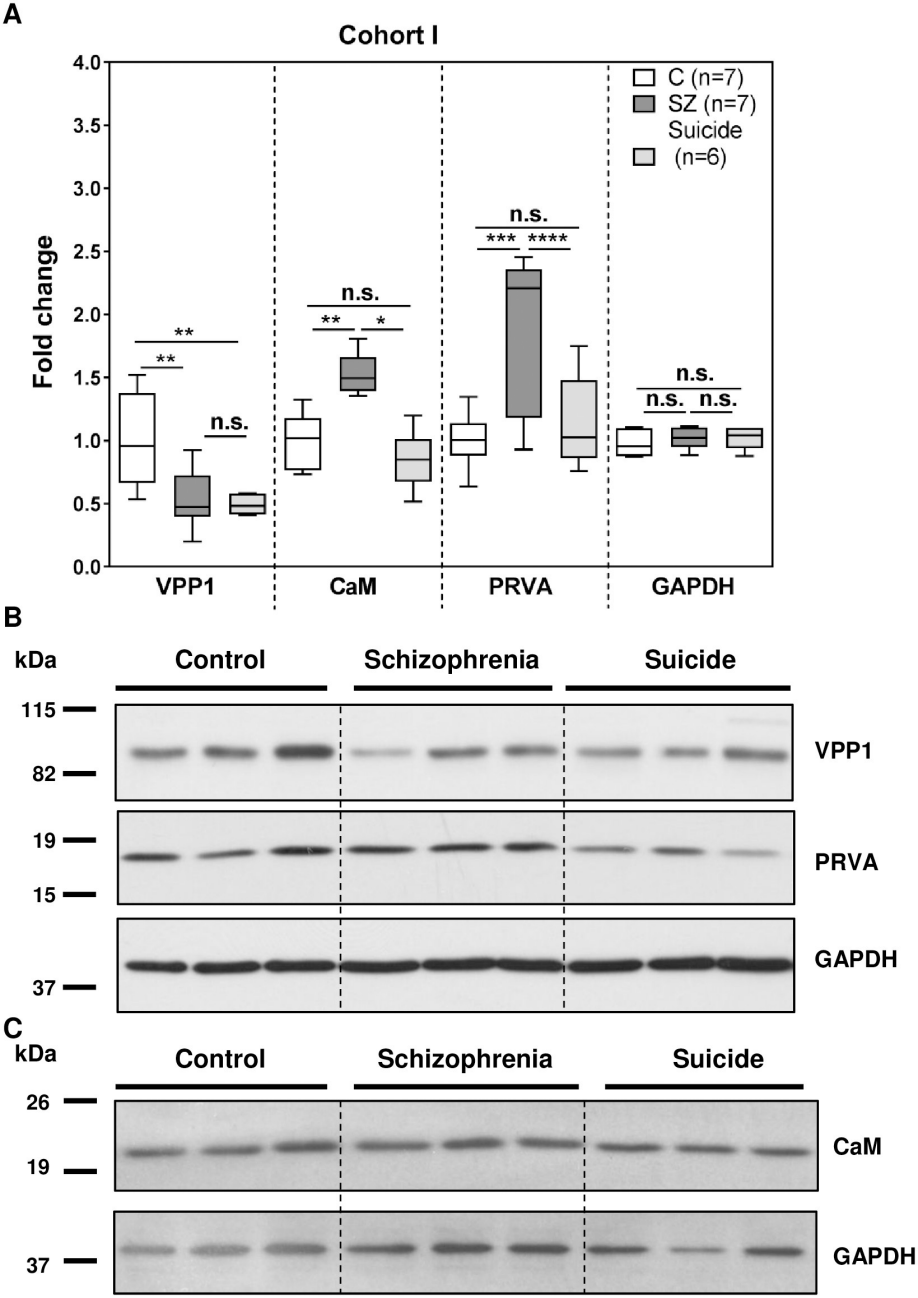
Validation analysis of hit candidate proteins by immunoblot in Cohort I. Protein extracts from samples of the post-mortem cerebellum of non-psychiatric control (C, n = 7), schizophrenia (SZ, n = 7) and non-schizophrenia suicide (n = 6) subjects (Table 1, Cohort I) were analysed by immunoblot for VPP1, PRVA, calmodulin (CaM) and GAPDH and quantified by densitometry. (A) Protein levels for each were normalized to GAPDH values and to the mean of the control samples. Each box plot represents the median, interquartile range and range of each group from at least two independent determinations. Statistical analysis was performed using the Kruskal-Wallis test for differences between groups (VPP1: p = 0.0095; PRVA: p = 0.0011; CaM: p = 0.0270) and the t test for comparison between the indicated groups. (n.s.-not significant, *p<0.05, **p<0.01, ***p<0.001, ****p<0.0001). (B) Representative Western blot images for VPP1, PRVA and GAPDH in 3 non-psychiatric control individuals, 3 schizophrenia subjects and 3 non-schizophrenia suicide controls. (C) Representative Western blot images for CaM and GAPDH in 3 non-psychiatric control individuals, 3 schizophrenia subjects and 3 non-schizophrenia suicide control. See S1 Raw images for complete western blot images.

We further characterized VPP1, calmodulin and parvalbumin by immunoblot in a cohort consisting of 7 schizophrenia subjects, 7 matched control subjects and 6 matched non-schizophrenia suicide subjects (Table 1, Cohort I). No differences in age, post-mortem delay and pH were found between comparison groups (Table 1, Cohort I). All candidate levels were referred to GAPDH levels, which were not significantly different between the groups (Fig 1A, 1B and 1C). We found that VPP1 protein levels were again significantly decreased [t = 2.809, df = 12, p = 0.0079; Mean ± SEM: control (C) = 1.000 ± 0.143, SZ = 0.526 ± .0.089] and calmodulin and parvalbumin levels were also significantly increased in the schizophrenia group (calmodulin [t = 3.724, df = 12, p = 0.0029; Mean ± SEM: C = 1.000 ± 0.083, SZ = 1.918 ± 0.232], parvalbumin [t = 4.964, df = 12, p = 0.0003; Mean ± SEM: C = 1.000 ± 0.0.086, SZ = 1.524 ± 0.060] (Fig 1A, 1B and 1C). In addition, we found significant differences between the schizophrenia group and the non-schizophrenia suicide group in the fold changes for calmodulin and parvalbumin, which in the suicide group showed similar levels to the control group [calmodulin [t = 2.708, df = 11, p = 0.0204; Mean ± SEM: SZ = 1.918 ± 0.232, suicide (SC) = 1.141 ± 0.149], parvalbumin [t = 6.129, df = 11, p < 0.0001; Mean ± SEM: SZ = 1.524 ± 0.060, SC = 0.849 ± 0.096] (Fig 1A, 1B and 1C). However, the protein levels of VPP1 were not significantly different in the non-schizophrenia suicide group compared to the schizophrenia group [t = 0.3354, df = 11, p = 0.7436; Mean ± SEM: SZ = 0.526 ± 0.089, SC = 0.492 ± 0.032] (Fig 1A, 1B and 1C). Furthermore, we analysed the influence of other demographic, clinical, and tissue-related variables in the differences found in the schizophrenia group compared to control subjects. None of our molecular measures showed any association with other variables of the study with the exception of parvalbumin, which showed an increase in the antipsychotic treated group compared to drug-free subjects (Table 4, Cohort I; Dunn’s Test p<0.05), indicating that the increase observed for parvalbumin in schizophrenia could be due to the antipsychotic treatments in these subjects.

**Table 4:** Association analysis of other variables in Cohorts I and II.

**Cohort I**					
		**Age**	**PMD**	**pH**	**Toxicology**
		**r**	**r**	**r**	**K**
**C-SZ cohort (n = 14)**					
VPP1		0.077	-0.007	-0.226	5.655
CaM^a^^,^ ^b^		-0.064	-0.381	0.239	1.257
PRVA		0.112	0.006	0.071	**6.600**^**,**^^c^
**Cohort II**					
	**Age**		**PMD**	**pH**	**Arterial Hypertension**
	**r**		**r**	**r**	**T; df**
**SZ-C cohort II (n = 27)**					
CaM^a^	0.221		-0.171	-0.048	1.13; 25
PRVA	0.032		0.208	0.169	0.55; 25
	**Daily AP dose**^d^	**Age of onset**	**Duration of illness**	**Body mass index**	**Medication side-effects**^e^
	r	r	r’	r	r’
**SZ cohort II (n = 13)**					
CaM	-0.116	-0.099	0.124	-0.170	0.062
PRVA	0.162	-0.170	-0.028	-0.231	0.189

r, Pearson’s r for parametric variables; r’, Spearman’s correlation for non-parametric variables; PMD, post-mortem delay; C, control; SZ, schizophrenia; AP, antipsychotic.

^a^r of Spearman are shown for this variable.

^**b**^ An outlier was detected for calmodulin (CaM) and therefore excluded from the SZ-C analysis (CaM: C, n = 13, SZ, n = 13).

^c^p<0.05.

^d^Last chlorpromazine equivalent dose was calculated based on the electronic records of drug prescriptions of the patients.

^e^Tardive Dyskinesia assessed by Abnormal Involuntary Movement Scale;

K, Kruskal-Wallis; T, T-statistic; df, degrees of freedom; N/A, not applicable. Significant associations are indicated in bold.

**Fig 2:**
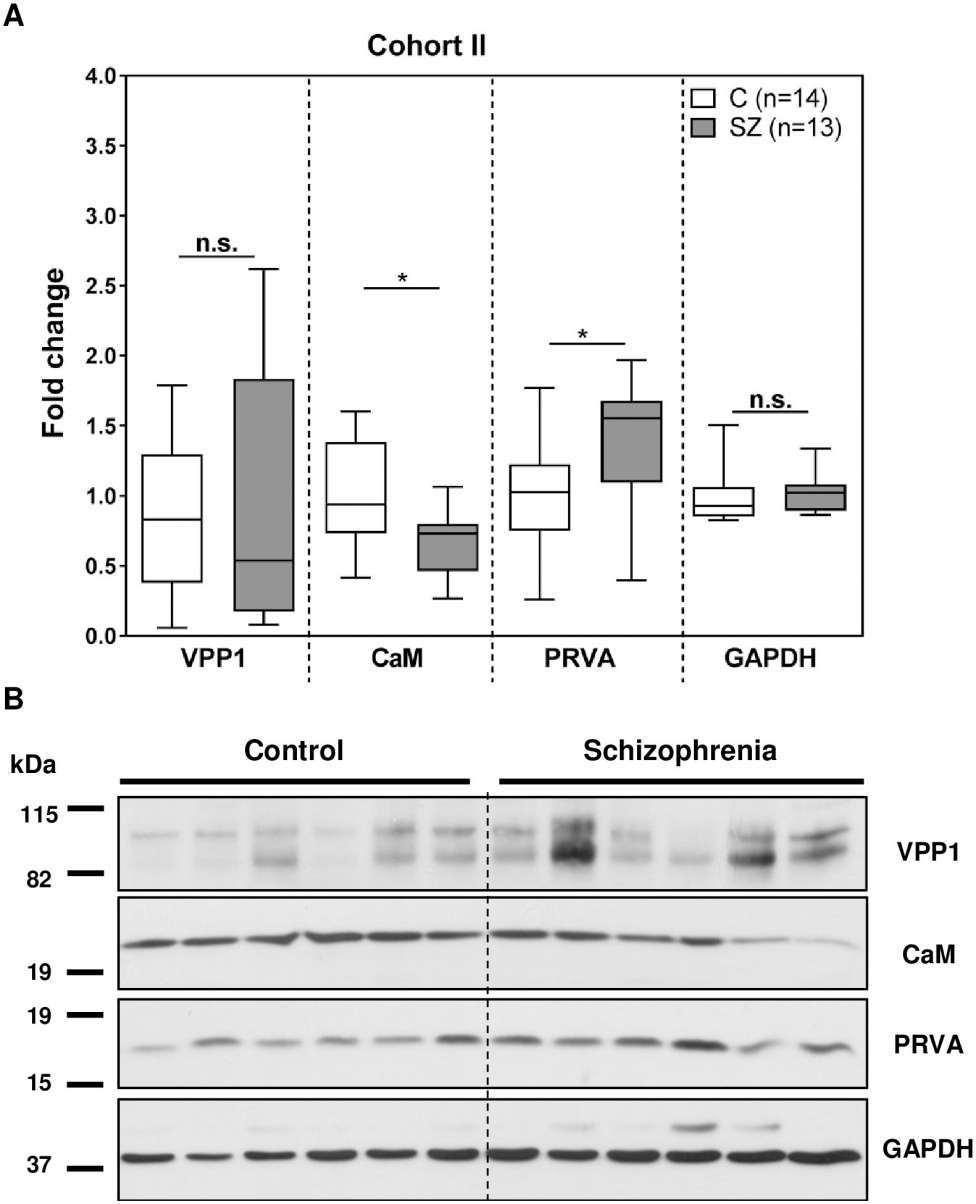
Validation analysis of hit candidate proteins by immunoblot in Cohort II. Protein extracts from samples of the post-mortem cerebellum of non-suicide chronic schizophrenia (SZ, n = 13) and control (C, n = 14) subjects (Table 1, Cohort II) were analysed by immunoblot for the same proteins as in Fig 1A and quantified by densitometry. (A) Protein levels for each protein were normalized to GAPDH values and to the mean of the control samples. Each box plot represents the median, interquartile range, and range of each group from at least two independent determinations. An outlier was detected for VPP1 protein values in the control group. Statistical analysis for comparison between case and control groups was performed using the t test for VPP1 and PRVA and the Mann-Whitney U test for CaM and GAPDH (n.s.-not significant, *p<0.05, ***p<0.001). (B) Representative Western blot images for VPP1, PRVA, CaM and GAPDH in 6 non-psychiatric control individuals and 6 non-suicide schizophrenia subjects from set 2. See S2 Raw images for complete western blot images.

We further characterized VPP1, calmodulin and parvalbumin protein levels by immunoblot in a larger, independent cohort of 13 non-suicide chronic schizophrenia subjects and 14 matched control individuals (Table 1, Cohort II). All candidate levels were referred to GAPDH levels, which were not significantly different between the groups (Fig 2A, 2B and 2C). We found that calmodulin and parvalbumin protein levels were significantly altered in the non-suicide schizophrenia group (calmodulin [U = 45.00, p = 0.0136; Mean ± SEM: C = 1.000 ± 0.3570, SZ = 0.6586 ± 0.2235], parvalbumin [t = 2.337, df = 25, p = 0.0139; Mean ± SEM: C = 1.000 ± 0.110, SZ = 1.380 ± 0.163]) (Fig 2A, 2B and 2C). VPP1 protein levels did not show a significant decrease in the schizophrenia group compared to the control group [t = 0.1384, df = 24, p = 0.4455; Median ± SEM: C = 0.844 ± 0.148, SZ = 0.884 ± 0.249] (Fig 2A, 2B and 2C). Association analysis of other variables in this cohort of chronic schizophrenia (age, post-mortem delay, pH, arterial hypertension, antipsychotic dose, age of onset, duration of the illness, body mass index, and medication side-effects) did not show any associations between calmodulin and parvalbumin protein levels and other variables (Table 4, Cohort II).

## Discussion

Proteomic screening of cerebellum samples from schizophrenia patients has helped to identify candidates with a putative relevant role in this disorder. The list of altered proteins in this region in suicide schizophrenia subjects provides the first step to analyse in depth some of the most robust candidates. A systematic validation of three candidates in two independent cohorts, one containing suicide individuals and another containing schizophrenia subjects that died of natural causes, revealed that only the two EF-hand calcium-binding proteins were altered in schizophrenia but not in non-schizophrenia suicide subjects, while the third candidate, a subunit of the proton pump ATPase, was linked to suicide behaviours and was not dysregulated in non-suicidal schizophrenia subjects. Thus, our findings propose that the EF-hand calcium-binding proteins parvalbumin and calmodulin could be involved in the pathophysiology of schizophrenia in the cerebellum by altering calcium-dependent signalling pathways involved in synaptic function. Moreover, this work provides evidence for the role of VPP1 in suicide behaviours with a possible impact on the synaptic vesicle cycle. Alterations in both the calcium-binding proteins in schizophrenia and in the proton pump ATPase in suicide behaviours could lead to a disruption in the cerebellar synaptic functioning of the neuronal circuits through different mechanisms as discussed below.

### Vacuolar proton ATPase pump

Here we describe a decrease in protein levels of the subunit of the vacuolar-type proton pump ATPase (VPP1) in suicide subjects with schizophrenia or other psychiatric disorders, but not in non-suicidal elderly schizophrenia patients, suggesting that this protein may be involved in suicide behaviours rather than in schizophrenia. Another report has also found a decrease in VPP1 in younger schizophrenia subjects in the anterior cingulated cortex using the Stanley Medical Research Institute’s (SMRI) Array Collection (http://www.stanleyresearch.org), which included 4 suicides out of 15 subjects [30]. In line with our results, VPP1 has been reported in a list of genes differently expressed in the frontal cortex in subjects with major depression that committed suicide [31]. VPP1 is part of the large multi-subunit complex of the vacuolar proton pump ATPase. This complex provides a proton motive force and is involved in many cellular and physiological functions (reviewed in [32]). V^+^H-ATPase pumps are composed of two functional components, the cytoplasmic V1 component with the catalytic activity for ATP hydrolysis and the V0 component, which forms a membrane embedded component that is required for proton translocation across the membrane, which occurs through the a subunit (reviewed in [32]). The VPP1 subunit is highly expressed in neuronal cells. This subunit is a major component of synaptic vesicles and provides the pH gradient and membrane potential required for neurotransmitter accumulation in the initial phase of the synaptic vesicle cycle [33,34]. Later studies provided evidence that subunit a of the V0 domain is also involved in synaptic vesicle release by mediating the membrane fusion events downstream of the t-SNARE docking of vesicles in a calcium/calmodulin-dependent manner [35,36]. Indeed, the VPP1 orthologue in fly neurons directly interacts with calmodulin, another protein that we found altered in schizophrenia (see above), and this interaction is required for recruiting calmodulin to synapses and for the viability function of VPP1 [35,37]. In addition, neurons lacking VPP1 in *Drosophila melanogaster* and *Caenorhabditis elegans* accumulate vesicles in the synaptic terminals, supporting the role of this protein in neurotransmitter release into the synaptic cleft [35,38,39]. Thus, the reduction of VPP1 that we found in the cerebellum in suicide subjects could be impairing normal neurotransmitter accumulation in synaptic vesicles and their subsequent release into the synaptic terminals. A limited synaptic response of the cerebellar neurons could contribute to the altered functioning of cerebellar circuits in suicide behaviours.

In agreement with this idea of altered synaptic activity in suicide are the findings of multiple studies. The regulation of the expression of genes involved in neurotransmission and synaptic function has been linked to suicide behaviours in different psychiatric disorders, including schizophrenia and major depression [31,40–43]. For example, increased expression of the 5-hydroxytryptamine (5-HT) receptor type 2A has been reported in the amygdala of suicide subjects together with increased 5-HT utilisation [44–46] as well as in other brain areas such as the hippocampus and the prefrontal cortex [47,48], suggesting a widespread dysregulation of the serotonin system in suicide subjects, which has led to numerous studies of serotonin function in the context of suicidality [49,50]. Thus, changes in intracellular proteins involved in neurotransmitter trafficking are likely to have a widespread effect on this neurotransmitter system. Interestingly, the cerebellum has been described as one of the neural routes altered in suicide behaviours related to monoaminergic signal transduction [51]. There is evidence of altered subcellular localisation of 5-HT2A receptors in Purkinje cells as well as an increase in white matter in the cerebellum in schizophrenia [52], suggesting a possible compensatory mechanism for compromised 5-HT synthesis or release in this region. Some reports have also linked reduced cerebellar activity and reduced grey matter with the ideation of suicide or suicide attempt, respectively [53,54]. Thus, the reduction of VPP1 observed in our study in suicide subjects in the cerebellum could alter the refilling of monoaminergic neurotransmitters (5-HT mainly) into synaptic vesicles and their release in cerebellar circuits. Moreover, it might mediate the decreased activity reported in this area in suicide behaviours.

### EF-hand calcium-binding proteins

Calcium homeostasis has been suggested to be disrupted in schizophrenia [55,56]. In our study, we found altered levels of two proteins that are sensitive to changes in the intracellular concentrations of calcium and that play a key role in signalling transduction and synaptic functioning.

#### Calmodulin

Calmodulin (CaM) belongs to the large family of EF-hand calcium-binding proteins. Here, we report an increase in calmodulin levels in the cerebellum in schizophrenia in the chronic SZ group with a mean age of 44 years, while calmodulin was found to be downregulated in a late elderly chronic schizophrenia cohort (mean age of 74 years). A previous study in an early elderly chronic schizophrenia cohort (mean age of 68) also reported a downregulation of calmodulin protein levels in different brain areas [57], while another study of chronic schizophrenia (mean age 67) reported an upregulation of calmodulin protein levels in nuclear-enriched cell samples from the corpus callosum and anterior temporal lobe [58]. In line with our study, these reports suggest a different regulation of calmodulin that may depend on age, brain region, or the cellular or subcellular locations under study. Calmodulin is the major calcium-binding protein present in the brain and acts as a calcium sensor, detecting and responding to biologically relevant changes in intracellular concentrations of calcium [59–61]. Calcium regulates calmodulin by changing its subcellular localization, promoting interaction with many proteins or by inducing conformational changes that allow the interaction and activation of specific targets and the subsequent triggering of a signalling cascade [59]. Calmodulin-dependent kinase II (CaMKII) has been proposed as a susceptibility gene in schizophrenia [62]. CaMKII is an important calmodulin effector in neurons that has been implicated in activity-dependent functions such as gene transcription, signalling and synaptic and dendritic development, maturation and function as well as in cognition [60,63]. The list of calmodulin effectors is extensive and includes plasma membrane calcium pumps, various ion channels, protein kinases and receptors, among others [59,61,64]. Calcium homeostasis is thus tightly regulated and features downstream of some of the mechanisms suggested to be altered in suicide subjects, such as an increase in 5-HT2A signalling, whose downstream intracellular cascades converge in the release of intracellular calcium. Thus, the calmodulin-dependent functions that could be dysregulated in schizophrenia in the cerebellum are wide, leading to an important impact on cerebellar circuit functioning and formation in accordance with the connectivity deficits and the neurodevelopmental hypothesis for schizophrenia [1,65]. In our study, we also found in suicide schizophrenia subjects a decrease in one interactor of calmodulin in flies, the orthologue of VPP1, which is required for recruiting calmodulin to synapses [37,66]. Indeed, the Ca^2+^-CaM regulation of V100 (VPP1 orthologue in flies) has been proposed as a positive regulator of the assembly of the SNARE complex on distinct vesicles and subsequent neurotransmitter release [37]. If this interaction is present in cerebellar neurons, this evidence could either suggest that the upregulation of calmodulin in the cerebellum in suicide schizophrenia subjects could be a mechanism to compensate for the possible lower abundance of calmodulin at the synapses due to a reduction in VPP1 levels or that it could be a compensatory mechanism for the lower formation/release of synaptic vesicles [37,66]. Further investigations will be needed to confirm this possibility. However, in chronic SZ patients that died from natural causes, calmodulin was decreased, suggesting a possible constitutive downregulation of calmodulin in chronic schizophrenia. In this context, VPP1 levels were not altered, raising the possibility that the recruitment of calmodulin to the synapse could be correct and the reduction of calmodulin could be impacting on other calmodulin-dependent functions in the cerebellum.

#### Parvalbumin

Here, we report an increase in parvalbumin in the cerebellum in schizophrenia independently of the mechanism of death. Parvalbumin, like calmodulin, also belongs to the large family of EF-hand calcium-binding proteins. However, parvalbumin is a calcium buffer protein, which are proteins essential for modulating calcium homeostasis in neurons and are implicated in the subtle regulation and timing of calcium signals pre- and post-synaptically [67–69]. Parvalbumin is a spatial and temporal regulator of calcium transients that modulates calcium pools and that is critical for synaptic plasticity, such as short-term facilitation [67,68]. Furthermore, it has been proposed that it could also regulate calcium signalling as a slow-onset calcium sensor in addition to being regulated by calcium [70]. Parvalbumin is expressed in subpopulations of GABAergic interneurons in the brain, which are considered more metabolically and electrically active than the neighbouring neurons and play an important role in the pathophysiology of schizophrenia [14,71,72]. Indeed, a decrease in the expression of parvalbumin in patients with SZ in different brain regions including the hippocampus and prefrontal cortex has been a consistent finding in human post-mortem studies [56,73] and in animal models of schizophrenia [74–77]. However, little is known about parvalbumin protein levels in the cerebellum of SZ patients. In the cerebellum, parvalbumin localizes to the axon, soma and dendrites of Purkinje, stellate, basket and a small proportion of Golgi cells [70]. Studies in parvalbumin-deficient mice suggest that this protein is required for normal locomotor activity in order to maintain the normal spontaneous arrhythmic and asynchronous firing pattern of Purkinje cells [78,79]. These mice also showed behavioural deficits linked to schizophrenia and autism, such as deficits in sensorimotor gating and novelty seeking and reduced social interaction and communication [80,81]. The increase in parvalbumin we observed in the cerebellum in schizophrenia could produce an imbalance in the regulation of intracellular calcium concentration and/or altering calcium-dependent signalling during synapse activity and this could have an impact on the behavioural changes observed in schizophrenia. The increase in parvalbumin could also reflect a change in the number of certain subpopulations of parvalbumin-positive neurons in the cerebellum in schizophrenia. However, a reduction in Purkinje neurons in the cerebellum has been reported in subjects with schizophrenia [72], suggesting that this possibility may be more likely due to the contribution from other types of parvalbumin-expressing neurons in the cerebellum. Further studies will be needed to elucidate these possibilities.

Functional neuroimaging studies show a predominantly hypoactivation of the cerebellum in schizophrenia [82,83]. Thus, based on our results, the altered EF-hand calcium-binding proteins found in the cerebellum could have an impact on synaptic transmission and underlie the reduced cerebellar activity observed in people with schizophrenia. Further studies are needed to investigate this hypothesis.

### Limitations

We acknowledge several limitations of our study. First, we used pooled samples in the proteomic screening. Although this type of design is a useful approach for detecting commonly altered pathways [84–88], it precludes the discovery of individual-specific changes or controlling for inter-individual variations. In our immunoblot analysis, the results obtained with pooled samples were recapitulated in individual samples from a similar cohort, suggesting that molecular changes in the cerebellum could be conserved across individuals. Second, the sample size of Cohort I is limited. Further analysis in a larger, independent cohort of samples including suicidal groups will be needed to explore how stable our findings are in other patients. Third, antipsychotic treatments could influence the results. To control for this variable, we have used the blood toxicology data in Cohort I and chlorpromazine equivalent daily dose data in Cohort II, showing that the increased expression of parvalbumin could be due to the antipsychotic treatments in Cohort I but not in Cohort II. Further pharmacological studies in cellular and animal models, as well as in drug-naive patients, will help to clarify the effect of antipsychotics on parvalbumin. Moreover, no information about the duration of the treatments was available for the schizophrenia groups. Thus, the effect of long-term treatments on our molecular variables could not be assessed. Fourth, the schizophrenia subjects used in our proteomic screening committed suicide, which could be influencing the findings observed. We have controlled for this possibility in the validation of candidates by including a group with subjects who committed suicide but with varying psychiatric diagnoses instead of schizophrenia and an independent cohort with non-suicide schizophrenia subjects that died from natural causes. These analyses have allowed us to detect that VPP1 alteration could be a common feature of suicide and suggest that this candidate may only be altered in suicide subjects rather than in schizophrenia. It may also be possible that alterations of these proteins could occur in other psychiatric disorders. Further studies of these candidates in anxiety and adjustment disorders will be helpful to explore this possibility. Last, the majority of the subjects included in both cohorts were male. Further studies in a cohort with equal representation of both genders would be of interest.

## Conclusions

In summary, our findings provide evidence for an upregulation of calcium-binding proteins in the cerebellum in schizophrenia together with a calmodulin downregulation in chronic schizophrenia in elderly subjects, suggesting an altered modulation of calcium signalling and calcium transients in synaptic responses in this region in schizophrenia with an impact on the normal functioning of cerebellar circuits. In addition, our study provides evidence for the alteration of VPP1 in the cerebellum linked to suicide behaviours, suggesting an involvement of defective synaptic vesicle cycle and the release of neurotransmitters in suicide behaviours.

## Supporting information

S1 Fig Experimental strategy for large-scale quantitative proteomic analysis and identification of differentially expressed proteins in cerebellum in schizophrenia. **(A)** Protein lysates from the post-mortem cerebellum of control (n = 4) and suicide schizophrenia (SZ, n = 4) subjects (Table 1) were processed as described in the experimental procedures section. In the analysis in pools, samples from the same group were pooled. In the analysis of individual samples from schizophrenia, a pool of controls was used to compare each individual sample of schizophrenia. Subsequently, protein database searches, peptide quantification and data analysis were performed as described in the experimental procedures section. A panel of 3 candidates from significantly regulated proteins was selected for further validation by immunoblot: first, in a pilot cohort which includes a group of non-schizophrenia suicide subjects (Table 1; Cohort I: SZ (n = 7), non-SZ suicide (n = 6), control (n = 7)) and then in a larger cohort of non-suicide chronic schizophrenia subjects (Table 1, Cohort II: non-suicide SZ (n = 13), control (n = 14)). **(B)** Distribution of the number of peptides quantified per protein from the data set of 2289 quantified proteins. **(C)** Normalized distribution of z-scores for confidently quantified proteins (>2 peptide sequences) (n = 1148). **(D)** Gene ontology classification of biological functions for non-significantly and significantly altered proteins with low variation in the cerebellum in SZ compared to the control. Transport (GO:0006810); Cell communication (GO:0007154); Signal transduction (GO:0007165); Metabolism (GO:0008152); Energy pathways (GO:0006091); Regulation of nucleobase, nucleoside, nucleotide and nucleic acid metabolism (GO:0019219); Cell growth and/or maintenance (GO:0008151); Protein metabolism (GO:0019538); Biological process unknown (GO:0000004).
(TIF)

S2 Fig Validation of hit candidate proteins by immunoblot in pools. Pooled protein extracts from samples of the post-mortem cerebellum of control (C, n = 4) and schizophrenia (SZ, n = 4) subjects from the *UPV/EHU brain collection* (S1 Table, a subgroup from Cohort I, Table 1) used in the proteomic screening were analysed by immunoblotting for VPP1, PRVA, calmodulin (CaM) and GAPDH. Protein levels for each hit were quantified by densitometry and normalized to GAPDH values and to the reference control sample. Images show representative immunoblots of a pool of control (left band, C) and a pool of schizophrenia (right band, SZ) subjects. Analysis was performed in duplicate. Bars represent mean ± standard deviation of the analysis of duplicates from two independent dissections, with the exception of PVALB, whose data are from a duplicate analysis of one dissection. Statistical analysis was performed using the t test (n.s.-not significant, **p<0.01, ***p<0.001).
(TIF)

S1 Raw images Validation analysis of hit candidate proteins by immunoblot in Cohort I. Protein extracts from samples of the post-mortem cerebellum of non-psychiatric control (C, n = 7), schizophrenia (SZ, n = 7) and non-schizophrenia suicide (n = 6) subjects (Table 1, Cohort I) were analysed by immunoblot for VPP1, PRVA, calmodulin (CaM) and GAPDH and quantified by densitometry. Images show uncropped images of the area of the membrane incubated with anti-VPP1, anti- parvalbumin (PRVA) (A), anti-CaM (B) and anti-GAPDH (A and B) of immunoreactivities of Fig 1. The samples shown in Fig 1 are delimited by a dashed line on the complete Western blot membranes. Arrows indicate the analysed band. X, sample not included in Fig 1. *, Non-analysed immunoreactivity.
(TIF)

S2 Raw images Validation analysis of hit candidate proteins by immunoblot in Cohort II. Protein extracts from samples of the post-mortem cerebellum of non-psychiatric control (C, n = 14) and schizophrenia (SZ, n = 13) subjects (Table 1, Cohort II) were analysed by immunoblot for VPP1, PRVA, CaM and GAPDH and quantified by densitometry. Images show uncropped images of the area of the membrane incubated with anti-VPP1, anti-PRVA, anti-CaM and anti-GAPDH of immunoreactivities of Fig 2. The samples shown in Fig 2 are delimited by a dashed line on the complete Western blot membrane. Arrows indicate the analysed band. X, sample not included in the Fig 2. *, Non-analysed immunoreactivity.
(TIF)

S1 Table Demographic, clinical and tissue-related features of cases used for quantitative proteomic analysis. Mean ± standard deviation or relative frequency are shown for each variable; PMD, post-mortem delay; SZ, schizophrenia; C, healthy control group; AP, antipsychotics; N/A, not applicable. ^1^Paranoid schizophrenia (n = 7). ^2^Mann-Whitney U is shown for non-parametric variables.
(DOCX)

S1 Dataset List of reliably quantified proteins in the cerebellum in schizophrenia. (Probability >90%).
(XLSX)

S2 Dataset Proteins significantly regulated in the cerebellum in schizophrenia, classified according to their biological function (FDR<0.1, coverage >5%). (XLSX)

S1 File Supplementary material and methods. (DOCX)
