# Supplementary figures and images for: Calcium-binding proteins are altered in the cerebellum in schizophrenia
Source: PLoS One. 2020 Jul 8;15(7):e0230400. doi: 10.1371/journal.pone.0230400 (PMC7343173; doi:10.1371/journal.pone.0230400)

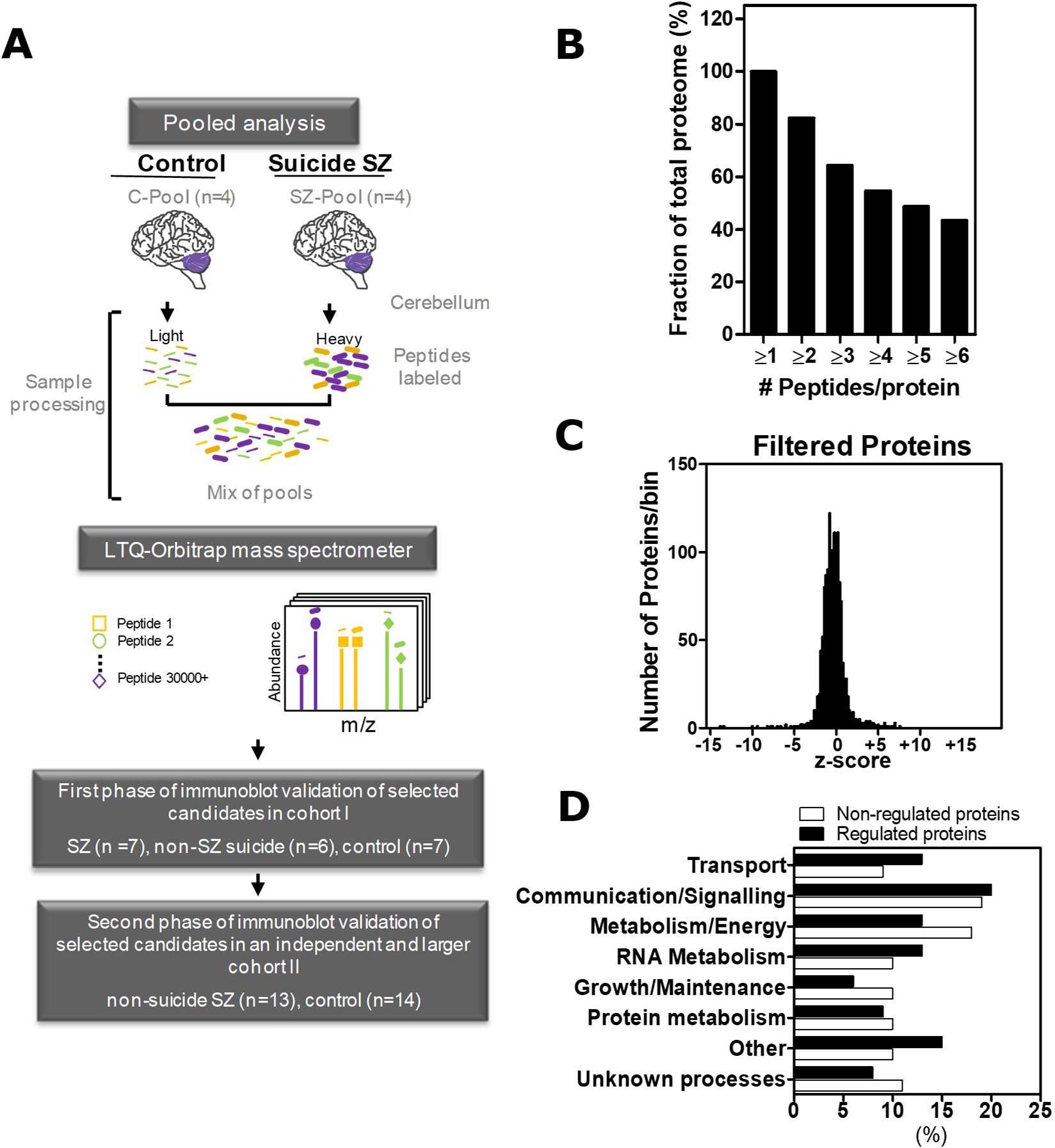

Supplement: S1 Fig — (A) Protein lysates from the post-mortem cerebellum of control (n = 4) and suicide schizophrenia (SZ, n = 4) subjects (Table 1) were processed as described in the experimental procedures section. In the analysis in pools, samples from the same group were pooled. In the analysis of individual samples from schizophrenia, a pool of controls was used to compare each individual sample of schizophrenia. Subsequently, protein database searches, peptide quantification and data analysis were performed as described in the experimental procedures section. A panel of 3 candidates from significantly regulated proteins was selected for further validation by immunoblot: first, in a pilot cohort which includes a group of non-schizophrenia suicide subjects (Table 1; Cohort I: SZ (n = 7), non-SZ suicide (n = 6), control (n = 7)) and then in a larger cohort of non-suicide chronic schizophrenia subjects (Table 1, Cohort II: non-suicide SZ (n = 13), control (n = 14)). (B) Distribution of the number of peptides quantified per protein from the data set of 2289 quantified proteins. (C) Normalized distribution of z-scores for confidently quantified proteins (>2 peptide sequences) (n = 1148). (D) Gene ontology classification of biological functions for non-significantly and significantly altered proteins with low variation in the cerebellum in SZ compared to the control. Transport (GO:0006810); Cell communication (GO:0007154); Signal transduction (GO:0007165); Metabolism (GO:0008152); Energy pathways (GO:0006091); Regulation of nucleobase, nucleoside, nucleotide and nucleic acid metabolism (GO:0019219); Cell growth and/or maintenance (GO:0008151); Protein metabolism (GO:0019538); Biological process unknown (GO:0000004). (TIF) [file pone.0230400.s001.tif]

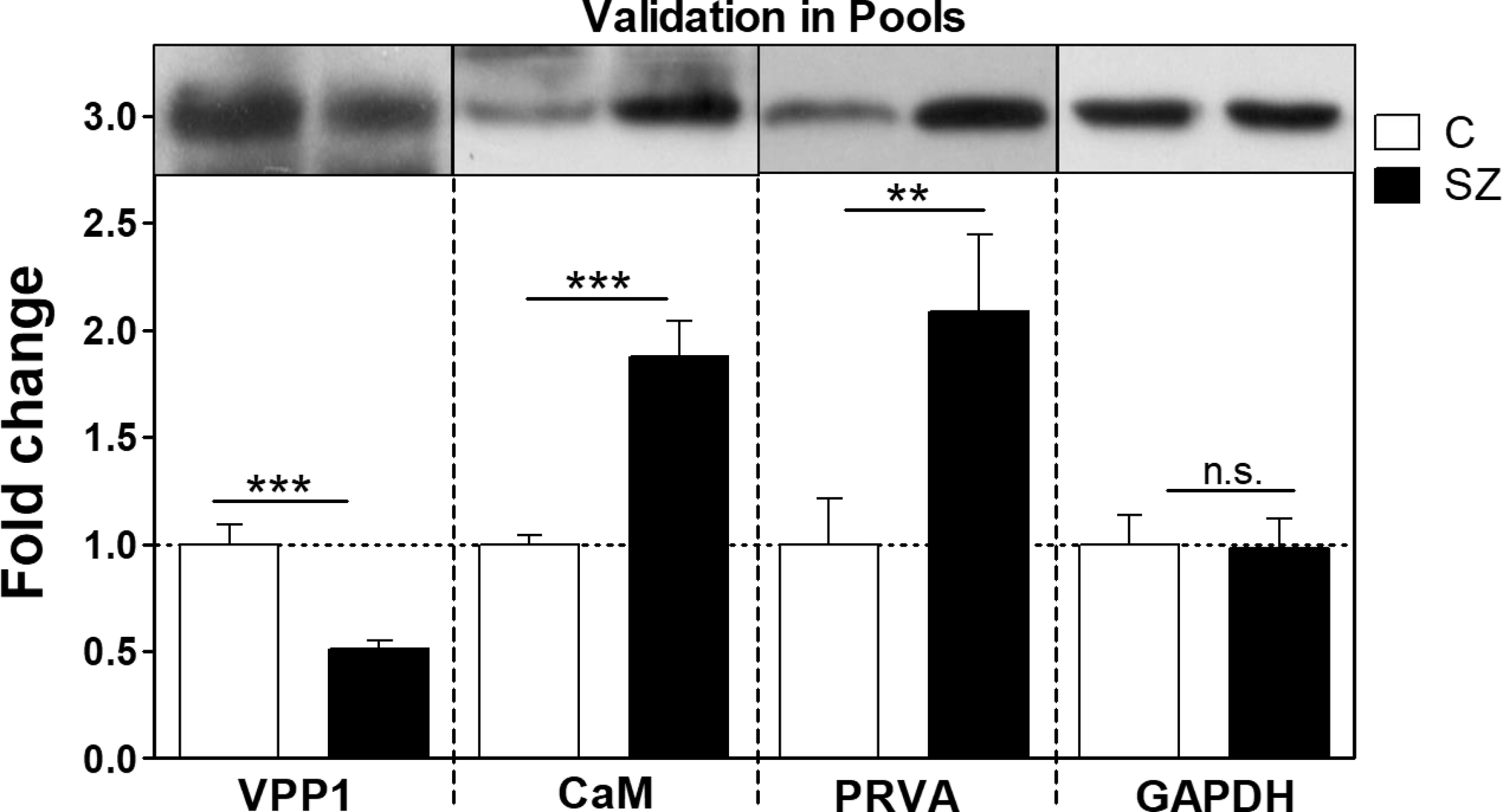

Supplement: S2 Fig — Pooled protein extracts from samples of the post-mortem cerebellum of control (C, n = 4) and schizophrenia (SZ, n = 4) subjects from the UPV/EHU brain collection (S1 Table, a subgroup from Cohort I, Table 1) used in the proteomic screening were analysed by immunoblotting for VPP1, PRVA, calmodulin (CaM) and GAPDH. Protein levels for each hit were quantified by densitometry and normalized to GAPDH values and to the reference control sample. Images show representative immunoblots of a pool of control (left band, C) and a pool of schizophrenia (right band, SZ) subjects. Analysis was performed in duplicate. Bars represent mean ± standard deviation of the analysis of duplicates from two independent dissections, with the exception of PVALB, whose data are from a duplicate analysis of one dissection. Statistical analysis was performed using the t test (n.s.-not significant, **p<0.01, ***p<0.001). (TIF) [file pone.0230400.s002.tif]

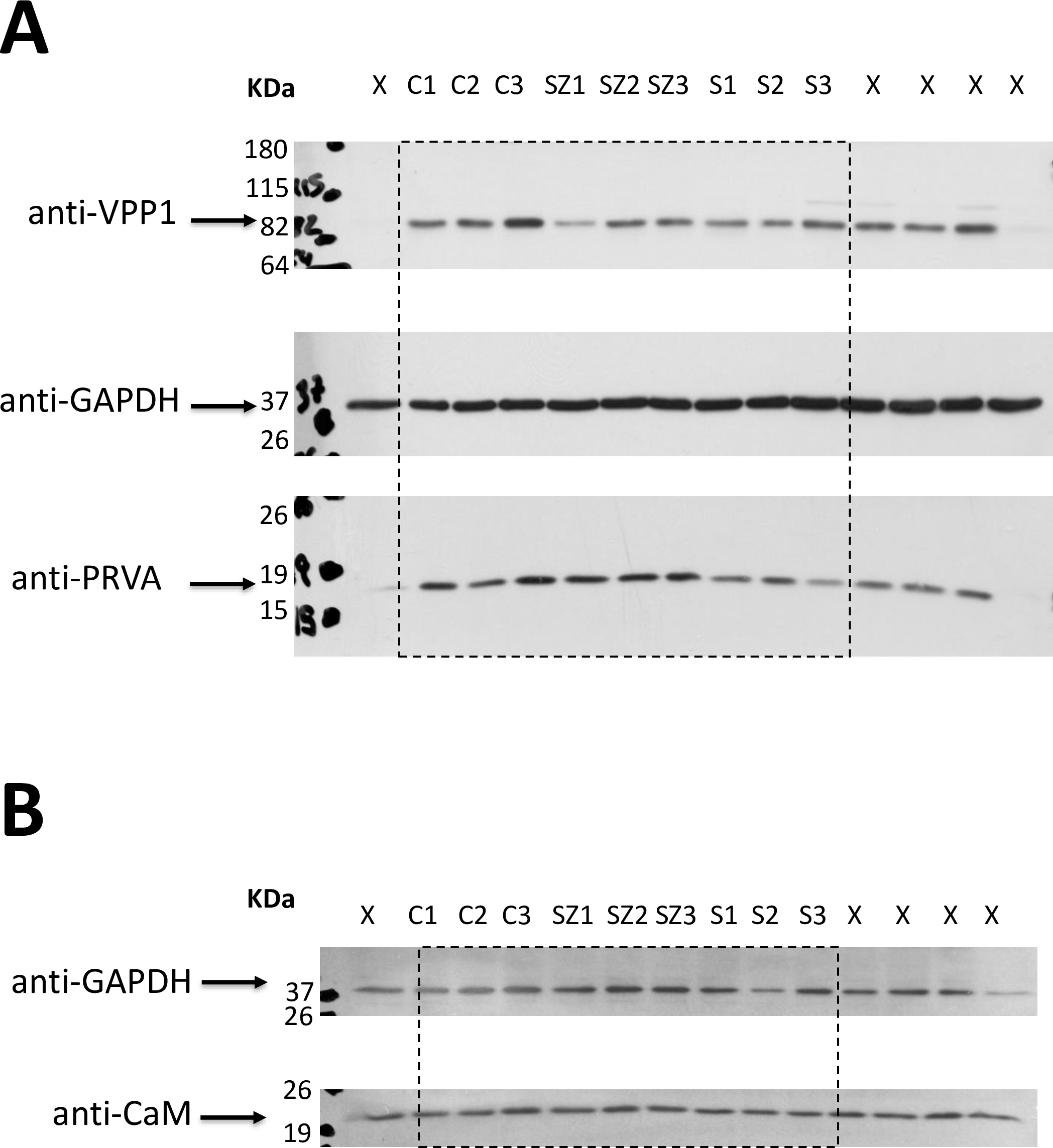

Supplement: S1 Raw images — Protein extracts from samples of the post-mortem cerebellum of non-psychiatric control (C, n = 7), schizophrenia (SZ, n = 7) and non-schizophrenia suicide (n = 6) subjects (Table 1, Cohort I) were analysed by immunoblot for VPP1, PRVA, calmodulin (CaM) and GAPDH and quantified by densitometry. Images show uncropped images of the area of the membrane incubated with anti-VPP1, anti- parvalbumin (PRVA) (A), anti-CaM (B) and anti-GAPDH (A and B) of immunoreactivities of Fig 1. The samples shown in Fig 1 are delimited by a dashed line on the complete Western blot membranes. Arrows indicate the analysed band. X, sample not included in Fig 1. *, Non-analysed immunoreactivity. (TIF) [file pone.0230400.s003.tif]

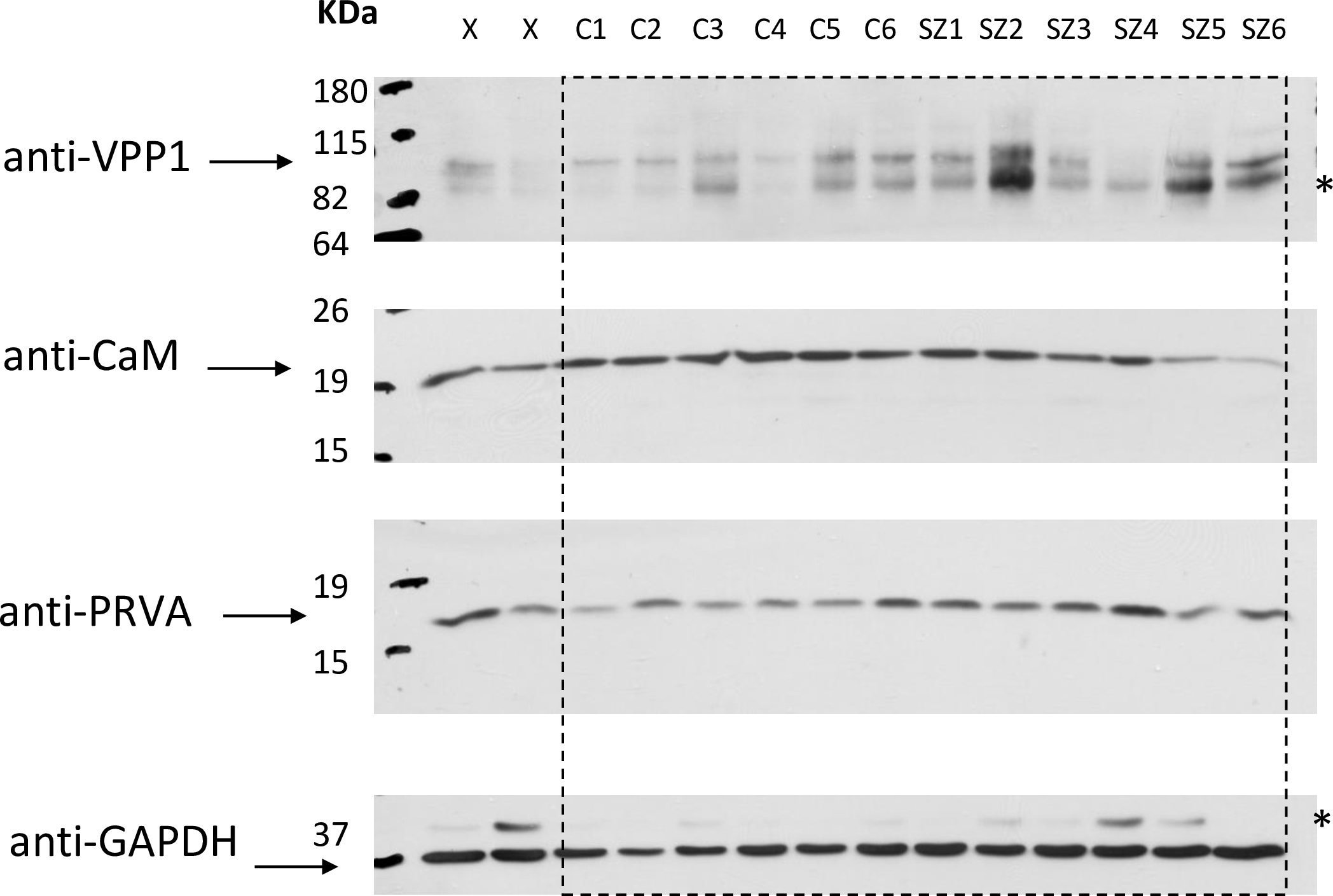

Supplement: S2 Raw images — Protein extracts from samples of the post-mortem cerebellum of non-psychiatric control (C, n = 14) and schizophrenia (SZ, n = 13) subjects (Table 1, Cohort II) were analysed by immunoblot for VPP1, PRVA, CaM and GAPDH and quantified by densitometry. Images show uncropped images of the area of the membrane incubated with anti-VPP1, anti-PRVA, anti-CaM and anti-GAPDH of immunoreactivities of Fig 2. The samples shown in Fig 2 are delimited by a dashed line on the complete Western blot membrane. Arrows indicate the analysed band. X, sample not included in the Fig 2. *, Non-analysed immunoreactivity. (TIF) [file pone.0230400.s004.tif]
