# Supplementary material for: Calcium-binding proteins are altered in the cerebellum in schizophrenia
Source: PLoS One. 2020 Jul 8;15(7):e0230400. doi: 10.1371/journal.pone.0230400 (PMC7343173; doi:10.1371/journal.pone.0230400)
Supplement: S1 Table — Mean ± standard deviation or relative frequency are shown for each variable; PMD, post-mortem delay; SZ, schizophrenia; C, healthy control group; AP, antipsychotics; N/A, not applicable. 1Paranoid schizophrenia (n = 7). 2Mann-Whitney U is shown for non-parametric variables. (DOCX) [file pone.0230400.s005.docx]

**S1 Table. Demographic, clinical and tissue-related features of cases used for quantitative proteomic analysis.**

| **Cohort I Subgroup (n=8)** | | | | |
| --- | --- | --- | --- | --- |
|  | **Schizophrenia^1^(n=4)** | **Control (n=4)** | **Statistic** | **p-value** |
| **Gender** |  |  |  |  |
| **Male** | 100% (n=4) | 100% (n=4) | N/A | N/A |
| **Age (years)** | 42 ± 11 | 42 ± 12 | ^2^7.50 | 1.000 |
| **PMD (hours)** | 8.25 ± 4.50 | 13.25 ± 7.59 | ^2^4.50 | 0.384 |
| **pH** | 6.68 ± 0.47 | 7.02 ± 0.29 | ^2^4.50 | 0.353 |
| **Toxicology** |  |  | N/A | N/A |
| **Atypical AP** | 50% (n=2) | N/A |  |  |
| **Other** | 50% (n=2) | 50% (n=2) |  |  |
| **Drug free** | N/A | 50% (n=2) |  |  |

Mean ± standard deviation or relative frequency are shown for each variable; PMD, post-mortem delay; SZ, schizophrenia; C, healthy control group; AP, antipsychotics; N/A, not applicable. ^1^Paranoid schizophrenia (n=7). ^2^Mann-Whitney U is shown for non-parametric variables.
