# Supplementary material for: Calcium-binding proteins are altered in the cerebellum in schizophrenia
Source: PLoS One. 2020 Jul 8;15(7):e0230400. doi: 10.1371/journal.pone.0230400 (PMC7343173; doi:10.1371/journal.pone.0230400)
Supplement: S1 File — (DOCX) [file pone.0230400.s008.docx]

**Supplementary materials and methods**

*In vitro isotope peptide labelling*

400µg of total extracts from four pooled control (100µg/each) and four pooled schizophrenia (100µg/each) lysates were reduced with 5mM dithiothreitol at 56ºC for 30 minutes and alkylated with 15mM iodoacetamide in 50mM Tris pH 8 in the dark at room temperature for 30 minutes. Each extract was digested with 5ng/µL trypsin in Tris 50mM, 1mM CaCl_2_ pH 8 at 37ºC for 16 h. Peptides were desalted by reversed-phase in a Sep-Pak C_18_ cartridge (100mg, Waters). Peptide mixtures were resuspended in 500 µl of 1M HEPES pH 7.5 and subjected to a reductive dimethylation reaction as described previously (Khidekel et al., 2007). Briefly, 20µl of a 600mM stock of NaCNBH3 or NaCNBD3 in water and 20µl of 4% aqueous formaldehyde-d0 (light labelling reaction) or 20µl of 4% aqueous formaldehyde-d2 (heavy labelling reaction) for the control or SZ samples respectively were added to the peptide solution, incubated for 10 minutes at room temperature and then quenched by adding 450µl of 10% Trifluoroacetic acid (TFA) (pH<3-4) and incubated for 1 hour. The light and heavy dimethylated peptide solutions were mixed 1:1. Peptides were desalted by reversed-phase in a Sep-Pak C_18_ cartridge. Peptide mixtures were resuspended in 5% acetonitrile (ACN) and 4% formic acid.

*Mass spectrometry analysis*

Dimethylated peptide mixtures were subjected to strong cation exchange chromatography on a polysulphoethyl A column. Twelve fractions were collected over 48 minutes in a gradient of KCl in 5mM potassium phosphate, 30% ACN, and dried by vacuum centrifugation. Peptides were resuspended in 1ml 0.1% trifluoroacetic acid, desalted by reversed-phase in a Sep-Pak C_18_ cartridge and dried by vacuum centrifugation. Peptide mixtures were resuspended in 5% ACN and 4% formic acid for LC-MS/MS analysis. Each peptide fraction was separated by reverse phase chromatography on a capillary column and analysed online on a hybrid linear ion trap Orbitrap (LTQ-Orbitrap XL, Thermo Scientific) mass spectrometer. For each cycle, one full MS scan acquired at high mass resolution (AGC target = 1 × 10^6^, maximum ion injection time = 1,000 ms) in the Orbitrap analyser was followed by 10 MS/MS spectra on the linear ion trap (AGC target = 5 × 10^3^, maximum ion injection time = 120 ms) for the ten most abundant ions. Fragmented precursor ions were dynamically excluded from further selection for 35s. Ions were also excluded if their charge was either <2 or unassigned. All spectra were acquired in centroid mode.

*Protein database searches, peptide quantification and data analysis*

Raw files were converted to mzXML format using ReAdW 2015.1.0 and default parameters. MS/MS spectra were searched against a concatenated target-decoy Uniprot human protein database (UP000005640 version 05-23-2017, n=71,567 target sequences) using the Comet search algorithm (version 2015025). Search parameters included full tryptic enzyme specificity with up to two missed cleavages permitted, mass tolerance of 50 ppm for the precursor and 1 Da for fragments ions, fixed modifications of carboxamidomethylation on cysteines (+57.02146) and dimethylation on lysines and peptide N-termini (+28.03130), and as variable modifications methionine oxidation (+15.99491) and the difference between heavy (6 hydrogen to deuterium) and light dimethyl on lysines and peptide N-termini (+6.03766). The RAW mass spectrometry proteomics data have been deposited in the ProteomeXchange Consortium via the PRIDE partner repository with the dataset identifier PXD008216 (Vizcaíno et al. 2016). Peptide matches were filtered to <1% false-discovery rate, the accepted threshold in the field, using the target-decoy database strategy and Percolator 3.1.2. Protein inference was carried out using Protein Prophet (contained in the Trans Proteomic Pipeline v4.8.0) and protein groups were filtered at ≥90 % probability score. Peptides were quantified using in-house software by peak-area integration, heavy and light peptide intensities were combined for every protein group and a log_2_ heavy/light ratio determined (Supplementary Data 1).

The log_2_ heavy/light ratio for each protein was transformed to a z-score for asymmetrical standard deviations of the main distribution as described previously (Graumann et al. 2008). A significance value (p-value) for each protein ratio was calculated from the complementary error function for the normalized distribution of the z-scores. To minimize the risk of type I errors and to estimate the proportion of true null hypotheses in our study, a False Discovery Rate (FDR) using the Benjamini and Hochberg method (Benjamini and Hochberg 1995) was also computed for all the p-values. The FDR threshold was set at 0.1. Significant proteins with consistent changes amongst peptides were then selected. Proteins were classified according to their biological function using the Human Protein Reference Database (HPRD-http://www.hprd.org). Up-regulated proteins were those with a z-score >0 and an FDR p-value <0.1 and down-regulated proteins were those with a z-score <0 and an FDR p-value <0.1. From these significantly altered proteins a panel of candidate proteins was then selected to be further validated by immunoblot following these criteria: (i) the change had been identified in more than 4 peptides; (ii) a greater than 2-fold increase or decrease.

**Supplementary references**

Behan, Á. T., C. Byrne, M. J. Dunn, G. Cagney, and D. R. Cotter. 2009. “Proteomic Analysis of Membrane Microdomain-Associated Proteins in the Dorsolateral Prefrontal Cortex in Schizophrenia and Bipolar Disorder Reveals Alterations in LAMP, STXBP1 and BASP1 Protein Expression.” *Molecular Psychiatry* 14(6):601–13.

Benjamini, Y. and Y. Hochberg. 1995. “Controlling the False Discovery Rate: A Practical and Powerful Approach to Multiple Testing.” *J Roy Stat Soc Ser B* 57:289–300.

Chan, M. K., T. M. Tsang, L. W. Harris, P. C. Guest, E. Holmes, and S. Bahn. 2011. “Evidence for Disease and Antipsychotic Medication Effects in Post-Mortem Brain from Schizophrenia Patients.” *Molecular Psychiatry* 16(12):1189–1202.

Clark, D., I. Dedova, S. Cordwell, and I. Matsumoto. 2006. “A Proteome Analysis of the Anterior Cingulate Cortex Gray Matter in Schizophrenia.” *Molecular Psychiatry* 11(5):459–70.

English, J. A., P. Dicker, M. Föcking, M. J. Dunn, and D. R. Cotter. 2009. “2-D DIGE Analysis Implicates Cytoskeletal Abnormalities in Psychiatric Disease.” *PROTEOMICS* 9(12):3368–82.

Föcking, M., P. Dicker, J. A. English, K. O. Schubert, M. J. Dunn, and D. R. Cotter. 2011. “Common Proteomic Changes in the Hippocampus in Schizophrenia and Bipolar Disorder and Particular Evidence for Involvement of Cornu Ammonis Regions 2 and 3.” *Archives of General Psychiatry* 68(5):477.

Föcking, M., L. M. Lopez, J. A. English, P. Dicker, A. Wolff, E. Brindley, K. Wynne, G. Cagney, and D. R. Cotter. 2015. “Proteomic and Genomic Evidence Implicates the Postsynaptic Density in Schizophrenia.” *Molecular Psychiatry* 20(4):424–32.

Graumann, J., N. C. Hubner, J. B. Kim, K. Ko, M. Moser, C. Kumar, J. Cox, H. Schöler, and M. Mann. 2008. “Stable Isotope Labeling by Amino Acids in Cell Culture (SILAC) and Proteome Quantitation of Mouse Embryonic Stem Cells to a Depth of 5,111 Proteins.” *Molecular & Cellular Proteomics* 7(4):672–83.

Khidekel, N., S. B. Ficarro, P. M. Clark, M. C. Bryan, D. L. Swaney, J. E. Rexach, Y. E. Sun, J. J. Coon, E. C. Peters, and L. C. Hsieh-Wilson. 2007. “Probing the Dynamics of O-GlcNAc Glycosylation in the Brain Using Quantitative Proteomics.” *Nature Chemical Biology* 3(6):339–48.

MacDonald, M. L., Y. Ding, J. Newman, S. Hemby, P. Penzes, D. A. Lewis, N. A. Yates, and R. A. Sweet. 2015. “Altered Glutamate Protein Co-Expression Network Topology Linked to Spine Loss in the Auditory Cortex of Schizophrenia.” *Biological Psychiatry* 77(11):959–68.

Martins-de-Souza, D., W. F. Gattaz, A. Schmitt, G. Maccarrone, E. Hunyadi-Gulyás, M. N. Eberlin, G. H. M. F. Souza, S. Marangoni, J. C. Novello, C. W. Turck, and E. Dias-Neto. 2009. “Proteomic Analysis of Dorsolateral Prefrontal Cortex Indicates the Involvement of Cytoskeleton, Oligodendrocyte, Energy Metabolism and New Potential Markers in Schizophrenia.” *Journal of Psychiatric Research* 43(11):978–86.

Martins-De-Souza, D., W. F. Gattaz, A. Schmitt, C. Rewerts, G. Maccarrone, E. Dias-Neto, and C. W. Turck. 2009. “Prefrontal Cortex Shotgun Proteome Analysis Reveals Altered Calcium Homeostasis and Immune System Imbalance in Schizophrenia.” *European Archives of Psychiatry and Clinical Neuroscience* 259(3):151–63.

Martins-de-Souza, D., W. F. Gattaz, A. Schmitt, C. Rewerts, S. Marangoni, J. C. Novello, G. Maccarrone, C. W. Turck, and E. Dias-Neto. 2009. “Alterations in Oligodendrocyte Proteins, Calcium Homeostasis and New Potential Markers in Schizophrenia Anterior Temporal Lobe Are Revealed by Shotgun Proteome Analysis.” *Journal of Neural Transmission* 116(3):275–89.

Martins-de-Souza, D., G. Maccarrone, T. Wobrock, I. Zerr, P. Gormanns, S. Reckow, P. Falkai, A. Schmitt, and C. W. Turck. 2010. “Proteome Analysis of the Thalamus and Cerebrospinal Fluid Reveals Glycolysis Dysfunction and Potential Biomarkers Candidates for Schizophrenia.” *Journal of Psychiatric Research* 44(16):1176–89.

Martins-de-Souza, D., A. Schmitt, R. Röder, M. Lebar, T. Schneider-Axmann, P. Falkai, and C. W. Turck. 2010. “Sex-Specific Proteome Differences in the Anterior Cingulate Cortex of Schizophrenia.” *Journal of Psychiatric Research* 44(14):989–91.

Nesvaderani, M., I. Matsumoto, and S. Sivagnanasundaram. 2009. “Anterior Hippocampus in Schizophrenia Pathogenesis: Molecular Evidence from a Proteome Study.” *Australian & New Zealand Journal of Psychiatry* 43(4):310–22.

Novikova, S. I., F. He, N. J. Cutrufello, and M. S. Lidow. 2006. “Identification of Protein Biomarkers for Schizophrenia and Bipolar Disorder in the Postmortem Prefrontal Cortex Using SELDI-TOF-MS ProteinChip Profiling Combined with MALDI-TOF-PSD-MS Analysis.” *Neurobiology of Disease* 23(1):61–76.

Pennington, K., C. L. Beasley, P. Dicker, A. Fagan, J. English, C. M. Pariante, R. Wait, M. J. Dunn, and D. R. Cotter. 2008. “Prominent Synaptic and Metabolic Abnormalities Revealed by Proteomic Analysis of the Dorsolateral Prefrontal Cortex in Schizophrenia and Bipolar Disorder.” *Molecular Psychiatry* 13(12):1102–17.

Pinacho, R., N. Villalmanzo, J. J. Meana, I. Ferrer, A. Berengueras, J. M. Haro, J. Villén, and B. Ramos. 2016. “Altered CSNK1E, FABP4 and NEFH Protein Levels in the Dorsolateral Prefrontal Cortex in Schizophrenia.” *Schizophrenia Research* 177(1–3):88–97.

Prabakaran, S., J. E. Swatton, M. M. Ryan, S. J. Huffaker, J. T. J. Huang, J. L. Griffin, M. Wayland, T. Freeman, F. Dudbridge, K. S. Lilley, N. A. Karp, S. Hester, D. Tkachev, M. L. Mimmack, R. H. Yolken, M. J. Webster, E. F. Torrey, and S. Bahn. 2004. “Mitochondrial Dysfunction in Schizophrenia: Evidence for Compromised Brain Metabolism and Oxidative Stress.” *Molecular Psychiatry* 9(7):684–97.

Saia-Cereda, V. M., J. S. Cassoli, A. Schmitt, P. Falkai, and D. Martins-de-Souza. 2016. “Differential Proteome and Phosphoproteome May Impact Cell Signaling in the Corpus Callosum of Schizophrenia Patients.” *Schizophrenia Research* 177(1–3):70–77.

Saia-Cereda, V. M., J. S. Cassoli, A. Schmitt, P. Falkai, J. M. Nascimento, and D. Martins-de-Souza. 2015. “Proteomics of the Corpus Callosum Unravel Pivotal Players in the Dysfunction of Cell Signaling, Structure, and Myelination in Schizophrenia Brains.” *European Archives of Psychiatry and Clinical Neuroscience* 265(7):601–12.

Saia-Cereda, V. M., A. G. Santana, A. Schmitt, P. Falkai, and D. Martins-de-Souza. 2017. “The Nuclear Proteome of White and Gray Matter from Schizophrenia Postmortem Brains.” *Molecular Neuropsychiatry* 3(1):37–52.

Schubert, K. O., M. Föcking, and D. R. Cotter. 2015. “Proteomic Pathway Analysis of the Hippocampus in Schizophrenia and Bipolar Affective Disorder Implicates 14-3-3 Signaling, Aryl Hydrocarbon Receptor Signaling, and Glucose Metabolism: Potential Roles in GABAergic Interneuron Pathology.” *Schizophrenia Research* 167(1–3):64–72.

Smalla, K. H., M. Mikhaylova, J. Sahin, H. G. Bernstein, B. Bogerts, A. Schmitt, R. van der Schors, A. B. Smit, K. W. Li, E. D. Gundelfinger, and M. R. Kreutz. 2008. “A Comparison of the Synaptic Proteome in Human Chronic Schizophrenia and Rat Ketamine Psychosis Suggest That Prohibitin Is Involved in the Synaptic Pathology of Schizophrenia.” *Molecular Psychiatry* 13(9):878–96.

Velásquez, E., F. C. S. Nogueira, I. Velásquez, A. Schmitt, P. Falkai, G. B. Domont, and D. Martins-de-Souza. 2017. “Synaptosomal Proteome of the Orbitofrontal Cortex from Schizophrenia Patients Using Quantitative Label-Free and ITRAQ-Based Shotgun Proteomics.” *Journal of Proteome Research* 16(12):4481–94.

Vizcaíno, J. A., A. Csordas, N. Del-Toro, J. A. Dianes, J. Griss, I. Lavidas, G. Mayer, Y. Perez-Riverol, F. Reisinger, T. Ternent, Q. W. Xu, R. Wang, and H. Hermjakob. 2016. “2016 Update of the PRIDE Database and Its Related Tools.” *Nucleic Acids Research* 44(D1):D447–56.
